# Effect of Polycyclic Aromatic Hydrocarbons Exposure on Sperm DNA in Idiopathic Male Infertility

**DOI:** 10.5696/2156-9614-9.21.190309

**Published:** 2019-03-14

**Authors:** Aziza A. Saad, Tarek Hussein, Amany El-Sikaily, Mohamed A. Abdel-Mohsen, El-Hassan Mokhamer, Amany I. Youssef, Jihan Mohammed

**Affiliations:** 1 Applied Medical Chemistry, Medical Research Institute, Alexandria University, Alexandria, Egypt; 2 Dermatology, Venereology and Andrology Department, Faculty of Medicine, Alexandria University, Alexandria, Egypt; 3 Marine Pollution Department, Marine Environment Division, National Institute of Oceanography and Fisheries, Alexandria, Egypt; 4 Molecular Biology lecturer, Zoology Department, Damnhour University, Damanhur, Egypt

**Keywords:** PAH, urinary metabolite, sperm DNA fragmentation, semen

## Abstract

**Background.:**

Biological mechanisms contribute to the relationship between polycyclic aromatic hydrocarbon (PAH) exposure and infertility in males by altering semen quality.

**Objectives.:**

The aim of the present study was to evaluate the impact of PAHs on male infertility using the sperm chromatin dispersion test (Halo sperm assay).

**Methods.:**

Sixty-six (66) infertile males under 45 years of age were examined for the determination of urinary metabolite and oxidative stress by measuring lipid peroxidation and antioxidant activity of glutathione and glutathione-s-transferase, as well as hormonal activity of follicle stimulating hormone (FSH), testosterone and prolactin and semen quality.

**Results.:**

There was an increased level of urinary metabolite of 1-hydroxy pyrene, 1-hydroxy naphthalene and 2-hydroxy naphthalene in the urine of the infertile group. In addition, elevated concentrations of malondialdehyde coincided with a decreased level of antioxidants, leading to oxidative stress in the infertile group. Semen samples showed 30% sperm deoxyribonucleic acid (DNA) fragmentation.

**Conclusions.:**

The data provide strong evidence of a statistical threshold for semen samples containing 30% sperm DNA fragmentation resulting in a reduced level of pregnancy success.

**Participant consent.:**

Obtained

**Ethics Approval.:**

Study approval was given by the ethics committee of Alexandria University (United States Department of Health and Human Services, institutional review board registration (IRB), IORG0008812 Medical Research Institute, expires 4/8/2019, OMB No: 0990-0279).

**Competing Interests.:**

The authors declare no competing financial interests.

## Introduction

Neuroendocrine regulation of physiological events leading to the production of mature sperm must occur to ensure the fertilization of mature oocytes, the development of normal embryos and delivery of viable offspring.^1^ The disruption of any part of this precise sequence of events leads to infertility or abnormal fetal growth if a sperm with a genetic defect fertilizes normal mature eggs. In developing countries, about 30% of couples experience some difficulty in trying to conceive. In about 50% of these couples, the male factor is partly responsible for the failure to conceive.^2^ For 25% of men assessed, no identifiable cause of abnormal semen analysis can be found. Polycyclic aromatic hydrocarbons constitute a group of toxic, lipophilic and endocrine-disrupting chemicals that are widely distributed in the environment.^3^ Reproductive endocrine disruptors can alter the hypothalamic/pituitary and testicular hormones that regulate spermatogenesis. Exposure to endocrine disrupting compounds can result in gonadal insufficiency and/or infertility.^4^ Biological mechanisms can contribute to the relationship between PAH exposure and infertility in males by altering semen quality. Some studies have suggested that PAHs and their metabolites may be hormonal active.

Pyrene is one of the most extensively produced PAHs in emissions from the combustion of petrol and diesel and the main source of PAHs in urban environments.^5^ Urinary 1-hydroxpyrene (1-OHP), a major metabolite of pyrene, is considered to be a good surrogate biomarker of total PAH exposure in human populations and is reported to reflect levels of PAH exposure from different sources such as ambient air, food and indoor air.^6–11^ Selevan et al. reported an association between air pollution episodes of elevated PAHs and increased asthenospermia, abnormal morphology and abnormal chromatin in human sperm.^12^ Another study found that the presence of PAH–deoxyribonucleic acid (DNA) adducts in sperm is associated with abnormal morphology.^13^ Xia et al. reported that exposure to PAHs at environmental levels is associated with increased risk of male idiopathic infertility.^14^ In addition, a significant association was found among men with higher 1-OHP level and sperm concentration and sperm number per ejaculum.^15^ In this context, in sperm, reactive oxygen species (ROS) can cause potential damage to plasma membranes and DNA integrity, motility and overall semen quality.^16^ Therefore, scavenging excess ROS is mandatory for normal spermatogenesis. Antioxidant enzymes and molecules such as superoxide dismutases, glutathione (GSH), and catalases are largely abundant in semen plasma and sperm cells.^17^ Most of these genes, including nuclear factor erythroid 2-related factor 2, superoxide dismutases, catalases, glutathione-s-transferase, glutathione peroxidase and nitric oxide synthase harbor sequence variants in humans, which in turn may cause male infertility in different ways.

The objective of the present study was to examine the association between urinary PAH metabolite levels and sperm DNA fragmentation in a volunteer sample of men with primary idiopathic infertility compared to fertile controls.

## Methods

1-Hydroxypyrene, 1-naphthol, β-glucuronidase from Helix pomatia (G7017), acetonitrile (HPLC grade), methanol (HPLC grade) and n-hexane (HPLC grade) were purchased from Sigma Chemical Co; (St. Louis, MO, USA). Halosperm G2 was developed by Halotech DNA for assessing sperm DNA fragmentation in humans.

**Abbreviations**
*1-OHP*: 1-hydroxpyrene
*AhR*: Aryl hydrocarbon receptor
*ANOVA*: Analysis of variance
*FSH*: Follicle stimulating hormone
*GSH*: Glutathione
*MDA*: Malondialdehyde
*ROS*: Reactive oxygen species

### Study population

A total of 66 male subjects under 45 years of age were divided into two groups. The first group included 15 healthy fertile males who had fathered at least one child in the last 2 years who served as control participants (group I). Group II was comprised of fifty-one male patients with primary idiopathic infertility, defined as failure to conceive after one year of unprotected intercourse and abnormal semen parameters (sperm concentration <15 million ml^−1^ with or without decreased sperm motility and/or increased abnormal morphology) according to the guidelines of the World Health Organization (WHO),^18^ with female partners who were healthy and had no obvious cause for delayed conception. They were selected from the andrology outpatient clinic of the Department of Dermatology, Venereology and Andrology, Main University Hospital, Faculty of Medicine, Alexandria University. According to the presence or absence of PAHs metabolite, this group was further subclassified into subgroups: Group II-a included 23 environmentally-exposed idiopathic infertile males and Group II-b included 28 occupationally-exposed idiopathic infertile males (drivers).

Written consent was provided by the study participants and study approval was given by the ethics committee of Alexandria University (United States Department of Health and Human Services, institutional review board registration (IRB), IORG0008812 Medical Research Institute, expires 4/8/2019, OMB No: 0990-0279).

### Sampling

Venous blood was collected from all enrolled subjects for the assay of whole blood reduced glutathione content, erythrocyte glutathione-s-transferase enzyme activity, serum malonaldehyde, serum testosterone, prolactin and follicular stimulating hormone (FSH).

Urine samples were collected from all subjects for detection of some urinary metabolites of PAHs, including 1-hydroxypyrene, 1-naphthol, 2-naphthol and urine creatinine.

Semen samples were collected from subjects for analysis according to World Health Organization criteria and DNA fragmentation by sperm chromatin dispersion test.^18^

### Statistical analysis

Data for the current study were statistically analyzed using Statistical Package for the Social Sciences version 20 (SPSS Inc., Chicago, IL, USA). Numerical data were expressed as mean ± SD, and the values for different variables of exposed and control subjects were compared.

#### Determination of blood glutathione content

The method used in the present study was based on the reduction of 5,5′ dithiobis- (2-nitrobenzoic acid) with GSH to produce a yellow compound.^19^ The reduced chromogen is directly proportional to GSH concentration and its absorbance was measured at 405 nm.

#### Determination of glutathione-s-transferase

Glutathione-s-transferase was determined by measuring the conjugation of 1-chloro- 2, 4-dinitrobenzene with reduced glutathione.^20^ The conjugation is accompanied by an increase in absorbance at 340 nm. The rate of increase is directly proportional to the glutathione-s-transferase activity in the sample.

#### Determination of serum lipid peroxide

The principle of this method is based on the reaction of lipid peroxides malondialdehyde (MDA) with thiobarbituric acid in an acidic medium forming a red pigment which extracted using n-butanol and measured at 530 nm.^21^ The concentration of MDA was expressed as nmol MDA/ml.

#### Determination of routine serum prolactin, testosterone and follicular stimulating hormone by chemiluminescent immunometric assay

The concentration of prolactin, testosterone and FSH were measured by Immulite 1000 prolactin, Immulite 1000 testosterone and Immulite 1000 FSH kits (Siemens Medical Solutions Diagnostics) based on a solid-phase two-site chemiluminescent immunometric assay.^22^,^23^

#### High-performance liquid chromatography identification and quantification of 1-hydroxypyrene in urine

1-hydroxypyrene was determined by enzymatic hydrolysis with 100 μl of β-glucuronidase and sulfatase for 11 hours at 37°C. and identified by high performance liquid chromatography.^24^

#### High-performance liquid chromatography identification and quantification of 1-naphthol and 2-naphthol in urine

Urine was hydrolyzed enzymatically with 30 μl of β-glucuronidase and sulfatase for 16 hours at 37°C in a shaking water bath.^25^

#### Semen analysis

A computer program (Mira Lab ISO 9001) was used to assist with determining sperm count, motility and morphology.^18^

#### Assessment of DNA fragmentation

Sperm DNA fragmentation was assessed using the Halosperm G2 test (Halotech DNA, SL, Madrid, Spain). The sperm chromatin dispersion test is based on a two main steps (i) controlled DNA denaturation and (ii) controlled protamine removal; this gives rise to partially deproteinized nucleoids in which the DNA loops expand, forming halos of chromatin dispersion.^26^

## Results

Table 1 illustrates the results of the urinary PAH metabolites in urine of the volunteer idiopathic infertile men and the fertile control group, where the mean concentration of 1-naphthol was 6 nmol/mmol creatinine and the mean concentration of 2-naphthol was 19.3 nmol/mmol creatinine. In addition, the concentration of 1-hydroxy pyrene was 1.7 nmol/mmol creatinine.

**Table 1:** Concentration of Metabolites of Polycyclic Aromatic Hydrocarbons in the Urine of the Exposed Idiopathic Infertile Groups

Sample no.	1-naphthol concentration	2-naphthol concentration	1-hydroxypyrene concentration	Total metabolite concentration
1			0.7	0.7
2	1.2	3.4	3.3	7.9
3	4.1	0.0	0.3	4.4
4	5.2	0.0	0.4	5.6
5	12.0	0.0	0.0	12
6	0.0	0.0	2.0	2.0
7	0.9	14.1	0.0	15.0
8	8.1	1.3	0.3	9.7
9	5.0	0.0	0.5	5.5
10	3.1	2.1	0.2	5.4
11	12.9	0.0	0.0	12.9
12	2.3	00	0.0	2.3
13	2.9	0.0	2.8	5.7
14	0.0	11.7	0.3	12.0
15	0.0	8.2	1.6	9.8
16	3.1	16.9	3.8	23.8
17	0.0	19.4	3.1	22.5
18	1.6	1.1	0.0	2.7
19	0.0	0.0	0.3	0.3
20	5.7	115.7	0.0	121.4
21	0.0	0.0	1.9	1.9
22	5.5	0.0	0.0	5.5
23	4.4	0.0	5.2	9.6
24	0.0	0.0	0.7	0.7
25	0.0	0.0	1.3	1.3
26	10.5	0.0	0.0	10.5
27	3.3	31.9	0.7	35.9
28	21.4	5.6	5.4	32.4
**Mean**	6.0	19.3	1.7	13.6
**SD**	5.11	31.71	1.68	23.06

Values are expressed in nmol/mmol creatinine.

The detection limit of 1-naphthol is 1.1 nmol/mmol creatinine, 1.1 nmol/mmol creatinine for 2-naphhol and 0.3 nmol/mmol creatinine for 1-hydroxypyrene.

A value of 0.0 indicates value was below the limit of detection.

Abbreviations: SD, standard deviation

Data on the malondialdehyde concentration in the sera of fertile controls and the two groups of idiopathic infertile men were also analyzed. The statistical analysis of variance (ANOVA) between groups showed that there was no significant difference between the control group and group II-a (idiopathic infertile patient group) in which the mean malondialdehyde concentration was 4.8±1.92 mM/ml. There was a statistically significant difference between the control group and group II-b, which had a mean concentration of 7.2±3.95 mM/ml (*Table 2*).

**Table 2:** Serum Malondialdehyde Content Across Study Groups

**Statistical Analysis**	**Control (n=15)**	**Group II-a (n=23)**	**Group II-b (n=28)**
Mean	3.8	4.8	7.2
Min – Max	2.0 – 6.7	2.0 – 8.9	2.3 – 18.0
SD	1.59	1.92	3.95
SE	0.410	0.399	0.747
p		0.252	0.003
p_1_			0.060

Values are expressed in nmol/ml.

Mean differences are significant when p, p_1_ < 0.05.

Comparison between groups was performed using the Kruskal-Wallis test.

Comparison between every two groups was done using the Mann-Whitney U test with Bonferroni correction.

p indicates level of significance when comparing the two idiopathic infertile groups.

p_1_ indicates level of significance when comparing group II-b (idiopathic infertile group) with the control group.

Abbreviations: SD, standard deviation; SE, standard error

Statistical ANOVA between groups for glutathione in the blood of the three study groups revealed a statistically significant difference between the control group and group II-a (idiopathic infertile men). There was also a significant decrease in glutathione between the control and group II-b (idiopathic infertile men) as shown in Table 3. There was also a slight decrease in the mean concentration of total glutathione (24.0 mg/dl, 23.2 mg/dl in group II-a and II-b, respectively) In the control group, the concentration of glutathione was 26.8 mg/dl. Glutathione levels in the exposed idiopathic group were significantly decreased compared with the control group (p≤0.005). Data were collected on the enzymatic activity level of glutathione-s-transferase in the erythrocytes of the controls and the two idiopathic infertile groups. Statistical ANOVA between groups showed that there was no significant difference between the control group and group II-a (idiopathic infertile men), but there was a statistically significant difference between the control group and group II-b idiopathic infertile men (p≤0.005) (*Table 4*).

**Table 3:** Whole Blood Glutathione Content Across Study Groups

**Statistical Analysis**	**Controls (n=15)**	**Group II-a (n=23)**	**Group II-b (n=28)**
Mean	26.8	24.0	23.2
Min – Max	20.3 – 32.0	19 – 28.2	18.4 – 30.0
SD	3.67	3.02	3.05
SE	1.018	0.659	0.576
p		0.048	0.005
p_1_			0.703

Values are expressed in mg/dl.

Mean differences are significant when p, p_1_ < 0.05.

Comparison between groups was done using the one-way ANOVA test.

Comparison between every two groups was done using the Scheffe post-hoc test.

p indicates level of significance when comparing the two idiopathic infertile groups with the control group.

p_1_ indicates level of significance when comparing group II-b (idiopathic infertile group) with the control group.

Abbreviations: SD, standard deviation; SE, standard error

**Table 4:** Blood Erythrocytes Glutathione-s-transferase Enzymatic Activity Levels Across Study Groups

**Statistical Analysis**	**Control (n=15)**	**Group II-a (n=23)**	**Group II-b (n=28)**
Mean	1.7	1.9	2.3
Min – Max	1.4 – 2.3	1.3 – 2.6	1.5 – 3.6
SD	0.28	0.38	0.43
SE	0.072	0.079	0.082
p		0.620	0.001
p_1_			0.002

Values are expressed in U/l

Mean differences are significant when p, p_1_ < 0.05.

Comparison between groups was done using the one-way ANOVA test.

Comparison between every two groups was done using the Scheffe post-hoc test.

p indicates level of significance when comparing two idiopathic infertile groups with control group.

p_1_ indicates level of significance when comparing group II-b (idiopathic infertile group) with the control group.

Abbreviations: SD, standard deviation; SE, standard error

In the statistical analysis of testosterone concentration in sera, there was a highly significant decrease between the control group and the two idiopathic infertile patient groups (*Table 5*). Statistical analysis of the FSH hormone level showed no significant difference between the control group and group II-a idiopathic infertile patient group (p≤0.015), but there was statistically significant difference between the control and the exposed idiopathic infertile patient group (p≤0.006) (*Table 6*).

**Table 5:** Serum Testosterone Levels Across Study Groups

**Statistical Analysis**	**Control (n=15)**	**Group II-a (n=23)**	**Group II-a (n=28)**
Mean	547.2	320.6	365.5
Min – Max	304 - 760	173 - 536	108 - 616
SD	170.06	94.54	137.78
SE	47.167	21.139	26.515
p		0.001	0.008
p_1_			0.389

Values are expressed in ng/dl.

Mean differences are significant when p, pi < 0.05.

Comparison between groups was done using the one-way ANOVA test.

Comparison between every two groups was done by the Games-Howell post-hoc test, p is the significant value when comparing the two idiopathic infertile groups with the control group, p_1_ is the significant value when comparing group II-b (idiopathic infertile group) with the control group.

Abbreviations: SD, standard deviation; SE, standard error

**Table 6:** Serum Follicle Stimulating Hormone Levels Across Study Groups

**Statistical Analysis**	**Control (n=15)**	**Group II-a (n=23)**	**Group II-b (n=28)**
Mean	2.7	4.0	5.1
Min – Max	1.6 – 3.9	1.4 – 8.0	1.6 – 10.3
SD	0.73	1.92	2.71
SE	0.201	0.418	0.564
p		0.189	0.006
p_1_			0.510

Values are expressed in mIU/ml.

Mean differences are significant when p, p_1_ < 0.05.

Comparison between groups was done using the Kruskal-Wallis test.

Comparison between every two groups was done using the Mann-Whitney U test with Bonferroni correction.

p indicates level of significance when comparing the two idiopathic infertile groups with the control group.

p_1_ indicates level of significance when comparing group II-b (idiopathic infertile group) with the control group.

Abbreviations: SD, standard deviation; SE, standard error

Statistical analysis of prolactin hormone levels showed a very high significant difference observed between the control group and group II-a (idiopathic infertile patients) (p≤0.003) and group II-b idiopathic infertile patients (p≤0.003) (*Table 7*).

**Table 7:** Prolactin Levels Across Study Groups

**Statistical Analysis**	**Control (n= 15)**	**Group II-a (n= 23)**	**Group II-b (n= 28)**
Mean	5.5	11.1	9.1
Min – Max	3.1 – 9.0	4.5 – 19.8	3.6 – 20.0
SD	1.93	3.56	4.40
SE	0.556	0.742	0.881
p		0.003	0.015
p_1_			0.081

Values are presented as ng/ml.

Mean differences are significant when p, p_1_ < 0.05.

Comparison between groups was done using the one-way ANOVA test.

Comparison between every two groups was done by the Games-Howell post-hoc test.

p indicates level of significance when comparing the two idiopathic infertile groups with the control group.

p_1_ indicates level of significance when comparing group II-b (idiopathic infertile group) with the control group.

Abbreviations: SD, standard deviation; SE, standard error

The ANOVA in sperm count showed a highly significant decrease in sperm count for the two idiopathic infertile patient groups compared to the control group (*Table 8*).

**Table 8:** Sperm Count Across Study Groups

**Statistical Analysis**	**Control (n= 15)**	**Group II-a (n=23)**	**Group II-b (n=28)**
Mean	87.5	26.8	23.7
Min – Max	67 – 120	0.7 – 77.9	1.0 – 72.0
SD	15.45	25.78	20.07
SE	3.989	6.253	3.793
p		0.003	0.003
p_1_			1.000

Values are presented as n×10^6^

Mean differences are significant when p, p_1_ < 0.05.

Comparison between groups was done using the Kruskal-Wallis test.

Comparison between every two groups was done using the Mann-Whitney U test with Bonferroni correction.

p indicates level of significance when comparing two idiopathic infertile groups with control group.

p_1_ indicates level of significance when comparing group II-b (idiopathic infertile group) with the control group.

Abbreviations: SD, standard deviation; SE, standard error

Statistical ANOVA of progressive motility (%) showed a very high significant decrease in the progressive motility (%) of the two idiopathic infertile patient groups compared to the control group (p≤0.001) (*Table 9*).

**Table 9:** Progressive Motility (%) Across Study Groups

**Statistical Analysis**	**Control (n= 15)**	**Group II-a (n=23)**	**Group II-b (n=28)**
Mean	69.5	29.5	39.1
Min – Max	59.7 – 80.0	0.0 – 63.8	0 – 65.8
SD	6.66	21.24	18.48
SE	1.719	5.285	3.695
p		0.001	0.001
p_1_			0.317

Mean differences are significant when p, p_1_ < 0.05.

Comparison between groups was done by one-way ANOVA test.

Comparison between every two groups was done by Games-Howell post-hoc test.

p indicates level of significance when comparing two idiopathic infertile groups with control group.

p_1_ indicates level of significance when comparing group II-b (idiopathic infertile group) with the control group.

Abbreviations: SD, standard deviation; SE, standard error

## Discussion

For 25% of study subjects, there was no identifiable cause of abnormal semen analysis. Reproductive endocrine dysfunction can alter the hypothalamic/pituitary and testicular hormones that regulate the formation of sperm. Gonadal insufficiency and/or infertility can occur as a result of exposure to endocrine disrupting compounds.^3^ The present results revealed a high level of urinary 1-hydroxy pyrene, 1-hydroxy naphthalene, 2-hydroxy naphthalene in the urine of the exposed infertile group. The PAH metabolites reflect a more accurate estimate of the actual PAH intake than the ambient air measurements because they estimate the internal dose.^26^ Several studies have shown that 1-OHP urine is a marker suitable for PAH exposure in different occupational groups?^27^,^28^ Urinary 1-OHP is a good biological index for the occupational exposure assessment of PAHs.^29^,^30^ Bouchard and Viau reported that urinary 1-OHP increased during the course of a working day.^31^ They identified 68 studies on occupational exposure to PAHs and recommended urinary 1-OHP be used as a tool for risk assessment of exposure to PAHs because 1-OHP excretion increases linearly with airborne concentrations of pyrene.^31^,^32^

The present results revealed the presence of urinary 1-hydroxy pyrene, 1-hydroxy naphthalene and 2-hydroxy naphthalene in the urine of the occupationally infertile group. A number of biological mechanisms may contribute to the relationship between exposure to PAHs and infertility in males by altering semen quality. Many studies examining the association between exposure to PAHs and sperm quality indicated that 1-OHP may affect male semen quality even at non-occupational exposure levels.^24^,^33^ Some studies have suggested that PAHs and their metabolites may be hormonal active.^34^ There is some evidence that PAHs can bind and stimulate the aryl hydrocarbon receptor (AhR), as in the male reproductive system, human sperm express abundant amounts of AhR and AhR translocator nuclear mRNA, and the presence of AhR in sperm provides a mechanism by which PAHs, dioxins and polychlorinated biphenyls can directly affect sperm function.^35^,^36^

After binding of PAHs or dioxins, AhR translocates in the nucleus to interact with foreign-responsive elements in the regulation of enzymes of the first and second phase, such as the P450 and glutathione-s-transferases, and is also involved in the metabolism of PAHs and dioxins.^36^,^37^

The above finding represents a good interpretation of the present results which showed a highly significant increase in the enzymatic activity of glutathione-s-transferase in the two idiopathic infertility groups. In addition to mediating the poisoning of environmental chemicals, AhR is also involved in several physiological functions independent of external chemical exposure. Several studies have demonstrated that the seminal vesicle declines in mice with severe age deficits. Based on the potential contribution of AhR to infertility in males, it is thought that changes in the activity of the AhR protein by genetic variation may modify the adverse effects of PAHs and contribute to individual sensitivity to infertility in males.^38^ The aryl hydrocarbon receptor regulates the response of many toxic aromatic hydrocarbons.^39^

It has been suggested that exposure to PAHs is associated with sperm DNA damage. Consistent with a previous study, it was observed that urinary increase of 2-hydroxynaphthalene levels were associated with tail augmentation (%).^40^,^41^ Interactive metabolites of PAHs may reach the testicles and epididymis, then interact with sperm DNA to form adducts.^42^ In addition, the compounds resulting from PAH oxidation have the ability to enter oxidation cycles, which increase the composition of ROS and thus cause DNA damage to sperm.^43–45^

Many reproductive toxic agents exhibit cell-type specificity resulting in increased DNA fragmentation in epididymal or ejaculated sperm. Thus, it appears that there are various mechanisms that result in DNA strand breaks in mature sperm. One of the primary DNA damaging agents is ROS.

The aforementioned findings were confirmed and were in agreement with the results of the present study which showed 32% - 40% DNA fragmentation in the estrogen receptor mediated DNA binding.

These facts were consistent with the present results which found a highly significant decrease in the concentration of testosterone in the serum of the two idiopathic infertile groups, in addition to a very significant decrease in the motility (%) and progressive motility (%) of the sperm of the two study groups. In addition, there was a positive correlation between testosterone concentration and total motility and progressive motility of sperm.

In the present study, the highly significant up-regulation of FSH level is believed to be associated with reduced negative feedback regulation triggered by decreased testosterone levels in blood circulation. The synergetic action of luteinizing hormone/testosterone and FSH is necessary for the initiation, maintenance and reinitiating of normal spermatogenesis. The current results also demonstrated a positive correlation coefficient between testosterone and total motility and progressive motility of sperm.

There was a significant increase in the level of prolactin in the serum of group II-b which was more pronounced than in group II-a. The high up-regulation of prolactin in the sera of group II-a may be one of the causes of infertility in this group. Hyperprolactinemia is known to suppress testosterone synthesis and male fertility through prolactin-induced hypersecretion of adrenal corticoids or by inhibiting the secretion of gonadotropin-releasing hormone through prolactin receptors on hypothalamic dopaminergic neurons.

Among the many factors that affect male fertility, oxidative stress has aroused considerable interest in recent years and it is believed that oxidative stress in the reproductive system has an impact on semen's capacity for fertilization.^46–48^

In the current study, a highly significant increase in the level of MDA in the sera of the idiopathic infertile group was observed. Malondialdehyde results also exhibited a negative correlation coefficient with testosterone, sperm count, motility and progressive motility. Increased formation of ROS has been correlated with reduction of sperm motility. The correlation between ROS and low movement can be explained by a series of events leading to a decrease in axonemal protein phosphorylation and sperm immobilization. Another hypothesis is that hydrogen peroxide can spread across membranes in cells and inhibit enzyme activity such as glucose-6-phosphate dehydrogenase, resulting in a decrease in the availability of nicotinamide adenine dinucleotide phosphate and the associated accumulation of oxidative glutathione and reduced glutathione. These changes can cause a decrease in the antioxidant defenses of spermatozoa, which ultimately leads to the peroxidation of membrane phospholipids.^49^,^50^

The protective action of GSH against ROS is facilitated by interaction with associated enzymes, such as glutathione peroxidase and glutathione reductase.^51^

The present data exhibited a significant decrease in the glutathione content in the blood of the environmentally-exposed infertile group and an even greater decrease in the blood of the occupationally-exposed infertile group.

Metabolism of PAHs by Leydig cells or metabolites produced elsewhere in the body and transferred to the testes may negatively affect the function of the Leydig cell. In this context, it should be noted that tissues in the male genitals and Leydig cells in particular metabolize PAHs.^52^ Consequently, benzo(a)pyrene metabolites have significantly reduced the ability of the Leydig cell to synthesize and release testosterone which may be due to aging caused by ROS.^53^,^54^ Peltola et al. have shown that ROS can damage critical components of the steroidogenic pathway in Leydig cells, including steroidogenic acute regulatory proteins.^55^

## Conclusions

The present study found a high level of urinary 1-hydroxy pyrene, 1-hydroxy naphthalene and 2-hydroxy naphthalene in the urine of the exposed infertile group. Additionally, compounds resulting from the oxidation of PAHs have the ability to enter redox cycles, which increase the formation of reactive oxygen species and thus cause sperm DNA damage. Elevated levels of lipid peroxidation and decreased antioxidant enzyme activity were accompanied with a decrease in testosterone.

In conclusion, the present findings indicate that the environmental and occupational levels of PAHs exposure adversely affect male semen quality. Future large-scale studies should incorporate additional biomarkers to generate a more accurate and full assessment of the effect of PAHs exposure on male fertility. This study is a pilot/exploratory work and further research is needed to control for other important factors related to PAH exposure and semen quality.
